# “Anæmia,” Physician’s Dose Book

**Published:** 1887-09

**Authors:** 


ANÆMIA: By Fredk. P. Henry, M. D., Professor of Clinical Medicine in the Philadelphia Polyclinic, &c. Reprinted from the Polyclinic. Philadelphia, P. Blackiston Son & Co., 1887.

This is an interesting little work, being an exhaustive treatise on an important subject, giving symptoms, exciting causes, anatomical characters, diagnosis, prognosis and treatment in general of aæmia. Then it goes into the varieties of anæmia, which are exhaustively treated also.

---

THE PHYSICIANS' DOSE AND SYMPTOM BOOK, 17th edition, containing the doses and uses of all the principal articles in the Materia Medica. By Joseph H. Wythe, M. D., Professor Histology and Microscopy, Cooper Medical College, San Francisco, Cal., P. Blakiston Son & Co., Philadelphia.

